# Evaluation of geriatric changes in dogs

**DOI:** 10.14202/vetworld.2015.273-278

**Published:** 2015-03-05

**Authors:** Soumyaranjan Pati, S. K. Panda, A. P. Acharya, S. Senapati, M. Behera, S. S. Behera

**Affiliations:** 1 Department of Veterinary Pathology, College of Veterinary Science and Animal Husbandry, Orissa University of Agriculture and Technology, Bhubaneswar, Odisha, India; 2 Department of Translational Research, Institute of Life Science, Bhubaneswar, Odisha, India; 3 Department of Veterinary Surgery and Radiology, College of Veterinary Science and Animal Husbandry, Orissa University of Agriculture and Technology, Bhubaneswar, Odisha, India

**Keywords:** canine, geriatric, haematology, serum biochemistry, urine analysis

## Abstract

**Aim:**

The present study has been envisaged to ascertain the old age for critical management of geriatric dogs considering the parameters of externally visible changes, haemato-biochemical alterations and urine analysis in geriatric dogs approaching senility.

**Materials and Methods:**

The study was undertaken in the Department of Veterinary Pathology in collaboration with Teaching Veterinary Clinic complex spanning a period of 1 year. For screening of geriatric dogs, standard geriatric age chart of different breeds was followed. The external characteristics such as hair coat texture, dental wear and tear, skin texture and glaucoma were taken as a marker of old age. Haematology, serum biochemistry and urine analysis were also included in the study.

**Results:**

External visible changes like greying of hair, dull appearance of hair coat, glaucoma, osteoarthritis, dental wear and tear were commonly encountered in the aged dogs. The haemoglobin, total erythrocyte count and packed cell volume showed a decreasing trend in the geriatric groups. Biochemical values like total protein, albumin, calcium level showed a decreasing trend while urea level with an increasing trend in geriatric dogs without any much alteration in serum glutamic-oxaloacetic transaminse, serum glutamic-pyruvate transaminase, cholesterol and creatinine. Physical examination of urine revealed yellow, amber, red, deep red color with turbidity and higher specific gravity. Chemical examination revealed presence of protein, glucose, ketone bodies, blood and bilirubin on some cases. The culture and sensitivity test of the urine samples revealed presence of bacteria with sensitive and resistance to some antibiotics.

**Conclusion:**

External visible changes are still the golden standard of determining the old age in dogs. Haemato-biochemical evaluation can be useful for correlating with the pathophysiological status of the animal. Biochemical analysis of urine can be employed rightly as kidney dysfunction is being major geriatric problem. Anaemia, jaundice, nephritis, hepatitis are the most common findings considered during old age.

## Introduction

Dog is considered as the first animal tamed and harnessed by man since the ancient times. It is always a priority of the dog owners for better health management of their pets at different periods of their lifespan. Geriatric or old age is the most crucial stage that needs special attention. Old age is manifested with change in behavioural pattern to different visible changes. Haematological, biochemical and urine analysis accompanied by structural and functional changes at the tissue or cellular levels are encountered during old age signalling the disease process. Signs of ageing are inevitable in older dogs with all these alterations indicating certain illness or diseases. Screening elderly dogs identified unrecognised and unreported health risk factors resulting in lifestyle modification and on-going monitoring, as well as showing age-related diseases which helps in early diagnosis and medical interventions to improve quality of life [1]. Veterinarians mostly rely on signs of age-related diseases as observed by the pet owners who usually don’t recognise cardinal signs as being important enough to report [2]. So screening of geriatric dogs is highly desirable and is widely recommended [3-5]. Therefore clinical management of senile dogs taking into consideration the above changes and illness are utmost important for the dog owners and clinicians.

External visible changes like denture wear, loss of elasticity of the skin, rough hair coat, etc. are common and conventional parameters for determining old age and primary screening of the geriatric dogs. At the advent of old age, haematological alterations like anaemia, lowered packed cell volume (PCV) and biochemical changes like decreased serum protein, increased cholesterol and urea level, etc. are commonly evident which can be correlated with dysfunction of different organs like liver, kidney, heart, spleen, nervous and musculoskeletal system etc. Some common disease conditions encountered in geriatric dogs are glaucoma, arthritis, skin diseases, arteriosclerosis, prostrate hyperplasia, fibrosis of liver and kidney. These systemic or organ level changes along with any diseases may lead to loss of functionality, efficiency and agility of the animal during this end part of the life.

The present study has been envisaged to ascertain the old age for critical management of geriatric dogs considering the parameters like external visible changes, haemato-biochemical alterations, urine analysis and the existence of any old age related diseases. It will be more useful in dogs where dog owners fail to keep the birth record of their pets, and also in case of stray dogs.

## Materials and Methods

### Ethical approval

Ethical approval was not necessary. All the animals under this research were clinical cases. However, all clinical cases were examined and treated as per standard examination and treatment procedure.

### Study area

The present study on clinico-pathological changes in geriatric dogs was undertaken in the Department of Veterinary Pathology and Teaching Veterinary Clinic Complex (TVCC), Bhubaneswar spanning a period of about 1 year from July 2012 to June 2013. The dogs were selected that were brought or referred for treatment to the TVCC, Department of Veterinary Pathology, Department of Veterinary Surgery and Radiology, State Government Veterinary hospitals and private clinics in and around Bhubaneswar city. For screening of geriatric dogs, standard geriatric age chart of different breeds as per Goldston [6] was followed. Also external visible characteristics such as hair coat texture, dental wear and tear, skin texture, glaucoma etc. were taken as a marker of old age. The dogs that have proper birth record were considered for our study. In total, 50 geriatric dogs were screened. Haematological examination of all the 50 cases, biochemical assay of 15 cases, urine analysis of 10 cases and bacteriological culture and sensitivity test of urine of same 10 geriatric cases were conducted by correlating with the geriatric stages of the dogs.

### Collection of the blood sample

Blood samples were collected from cephalic/recurrent tarsal vein after application of rectified spirit at the site. Approximately, 6 ml of blood was collected aseptically in a clean sterilized disposable syringe. About 2 ml blood was transferred to one ethylenediaminetetraacetic acid vial for haematological study. Rest amount of blood was transferred to one serum collection tube. After collection, the blood samples were immediately transferred to the laboratory for further analysis. Serum was separated from the blood by centrifuging at 2000 rpm for 10 min. The serum was collected carefully, kept in a sterilized collection tube and stored in the deep freezer for subsequent analysis of biochemical parameters. In 15 cases, it was possible to collect requisite amount of blood for serum collection to conduct biochemical assay. Precautionary measures were taken to prevent haemolysis.

### Collection of the urine sample

The urine samples were collected by both voluntary urination and catheterization from 10 geriatric dogs. In voluntary voiding, utmost care was taken to collect the urine aseptically in a sterile container without any external contamination. Catheterization was done by using No.5, No.6 and No.7 nasogastric feeding tube according to the size of the animal after proper restraining of the dog. Urine was collected aseptically in a sterile container. This method was considered as a better method among two as there is lesser chance of contamination.

### Haematology

Haematological parameters were studied using standard methods such as haemoglobin (Hb) by Sahli’s acid haematin method, total erythrocyte count (TEC) and total leukocyte count (TLC) by haemocytometer, differential leukocyte count by Leishman’s stain and PCV by Wintrobe’s haematocrit tube method. Mean corpuscular volume (MCV) and mean corpuscular Hb concentration (MCHC) were estimated by using formula as MCV in cubic microns=(PCV/red blood cells [RBC] in millions) ×10 and MCHC% = (Hb in g/100 ml/PCV) ×100.

### Serum biochemistry

Plasma glucose, triglycerides, cholesterol, calcium, magnesium, phosphorus, total protein, albumin, globulin, urea, creatinine, activity of aspartate aminotransferase and alanine aminotransferase, were estimated by spectrophotometer using commercial reagent kits.

### Examination of the urine sample

From the collected urine samples, 1/3^rd^ of volume of each case was sent for bacteriological culture and sensitive test. Remaining 2/3^rd^ of urine was used for routine and microscopic examination. Routine examination included the colour, transparency, specific gravity, pH, Bendict’s test for glucose, Rothera’s test for ketone bodies, Hay’s test for bile salt, Robert’s test for protein and Salkowitch’s test for calcium in the urine.

### Statistical analysis

Statistical analysis was done using Microsoft Excel spreadsheet.

## Results

In the present study, the average age of 50 geriatric dogs was 10.21±0.37 years while it was 9.30±0.26 years as per Goldston [6]. In our study the distribution of different geriatric dogs were Spitz (15, 30%) followed by Deshi (8, 16%), GSD (5, 10%), Labrador (4, 8%), Mixed (3, 6%), Dachsund (3, 6%), Greatdane (3, 6%), Pomerian (3, 6%), Rottweiler (3, 6%), Golden Retriever (1, 2%), Pug (1, 2%) and Beagle (1, 2%) out of total 50 dogs.

**Figure-1:**
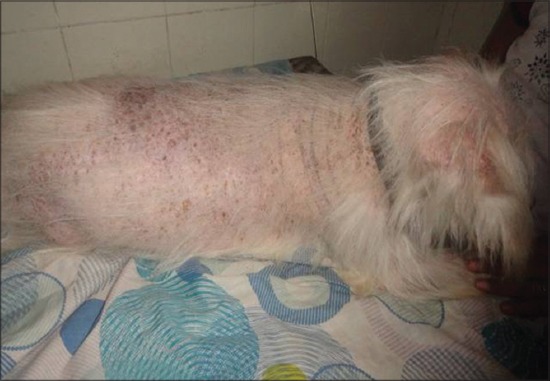
Around 90% alopecia in a geriatric dog

**Figure-2:**
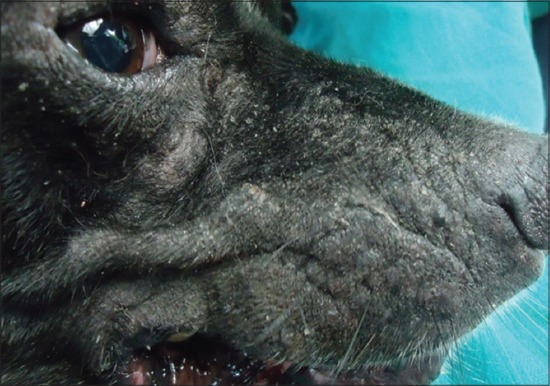
Loss of elasticity, wrinkling and thickening of skin on face region

**Figure-3:**
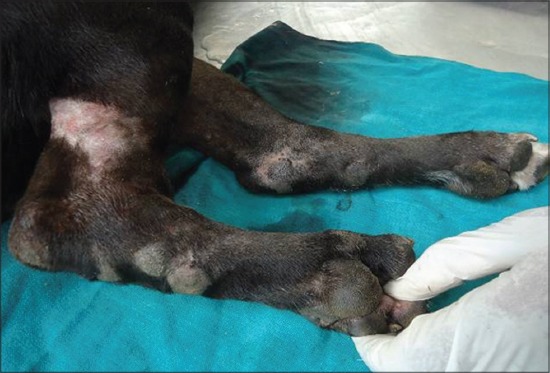
Thickening and callus formation on limb and food pad

**Figure-4:**
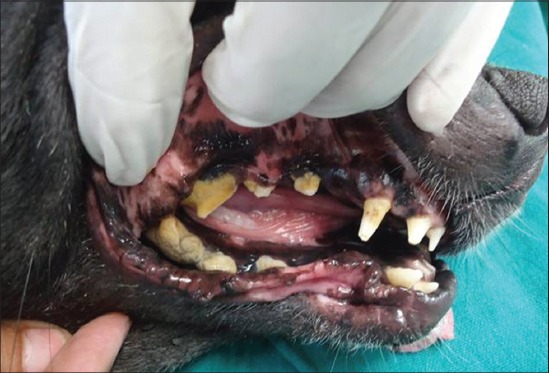
Dental wear and tear, tartar and yellowish discoloration of teeth

**Figure-5:**
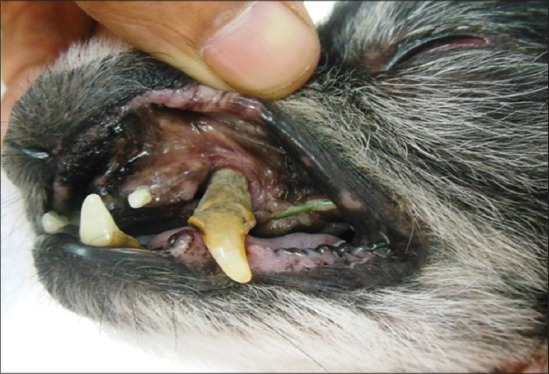
Tooth loosening with dental tartar in an old dog

**Figure-6:**
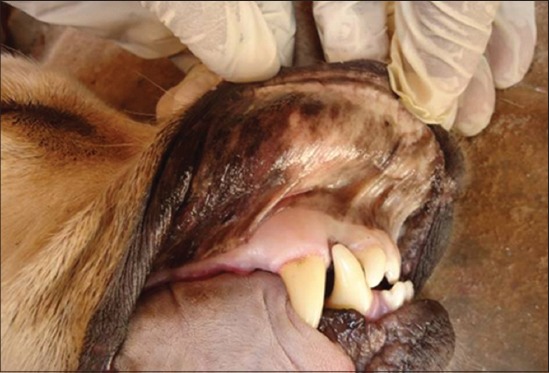
Pale mucous membrane in old age dog

Analysis of external visible changes showed presence of multiple features associated with old ages. External visible changes revealed findings like, graying of hair with dull appearance of skin and hair coat, dry skin and greying of hair around eye and muzzle, rough hair coat and alopecia (Figure-1), loss of elasticity of skin, wrinkling of skin (Figure-2), thickening of foot pad, formation of callus (Figure-3), dental wear and tear, dental tartars (Figure-4), gingivitis, broken tooth, loosen tooth, yellowish discoloration of teeth (Figure-5), halitosis, pale mucous membrane (Figure-6), glaucoma and limping due to osteoarthritis etc.

### Haematological examination

The aged dogs have an altered haematological profile. Alterations in different haematological parameters were depicted in Table-1. The hemoglobin profile in the geriatric group fluctuated from 5.6 g% to 12.2 g% with, the mean value of 9.77±0.17 g% that was lower than the normal value. TEC value had slightly decreased mean value at 5.21±0.12 million/mm^3^ with a minimum value 3.6 million/mm^3^ and maximum value 7 million/mm^3^. Mean and standard error value of PCV was found to be 32.88% and 5.44% with minimum and maximum value of 20% and 35% respectively. TLC value had slightly decreased mean value at 10094.20±1608.85/mm^3^ with minimum value 3000 and maximum value 85090/mm^3^ in old dogs. While analysing the differential count value, it was revealed that mean neutrophil count 67.23±1.25% was higher than the normal value. The value fluctuated from 27% to 89%. The eiosinophil and basophil percentage values were 1.66±0.22 and 0.04±0.03 that were within the normal physiological limit. The mean percentage value of lymphocyte and monocyte were 22.98±1.65 and 1.24±0.13 respectively which showed there was a decrease in value in both the parameters.

**Table-1:** Alteration in different haematological parameters

Parameters	Units	Mean±SE	Observation range
Hb	g/dl	9.77±0.17	5.6-12.20
TEC	×10^6^/µl	5.21±0.12	3.6-7
PCV	%	32.88±5.44	20-35
TLC	×10^3^/µl	10.09±1.60	3-85.09
Neutrophils	%	67.23±1.25	27-89
Eosinophils	%	1.66±0.22	0-7
Basophils	%	0.04±0.03	0-1
Lymphocytes	%	22.98±1.65	6-72
Monocytes	%	1.24±0.13	0-4

SE=Standard error, TLC=Total leukocyte count, PCV=Packed cell volume, Hb=Haemoglobin

### Biochemical examination

The aged dogs have an altered serum biochemical profile. The detailed biochemical parameters values were given on the Table-2. The mean value of glucose ranged between 73.811 and 172.904 with an average of 106.48±5.84. The mean calcium value in geriatric dogs ranged between 5.082 and 38.933 with an average of 9.53±2.15. The average phosphorus value was 3.72±0.31 ranging from 0.787 to 5.293. Magnesium concentration in serum in geriatric dogs hovered between 0.782 and 2.91 with mean having 1.93±0.17. Total protein in old dog’s varied between 2.95 and 5.62 with mean and standard error being 4.14±0.18. The average albumin value was 2.61±0.15 with a range of minimum 1.67 and maximum 3.84 which was lower than normal. The average cholesterol value was 228.24± 10.24 ranging from 165.74 to 301.35. The value of serum glutamic-oxaloacetic transaminse (SGOT) in the geriatric dogs varied from a minimum of 20.60 to a maximum of 53.07 with a mean value of 35.23±2.13. Similarly, serum glutamic-pyruvate transaminase (SGPT) value ranged from 13.97 to 54.3 with an average of 29.24±2.72. The value of urea and creatinine ranged between 18.481 to 45.93 and 0.571 to 2.363 respectively, and the average value was found 30.27±1.84 and 1.05±0.11 respectively. Total protein, calcium level showed a decreasing trend while urea level with an increasing trend in geriatric dogs.

**Table-2:** Alteration in different biochemical parameters

Parameters	Units	Average±SE	Observation range
Glucose	(mg/dl)	106.48±5.84	73.81-172.90
Calcium	(mg/dl)	9.53±2.15	5.082-38.93
Albumin	(mg/dl)	2.61±0.15	1.67-3.84
Total protein	(g/dl)	4.14±0.18	2.95-5.62
Magnesium	(mEq/L)	1.93±0.17	0.78-2.91
Cholesterol	(mg/dl)	228.24±10.24	165.74-301.35
Phosphorous	(mg/dl)	3.72±0.31	0.78-5.29
SGPT	(IU/L)	29.24±2.72	13.97-54.3
SGOT	(IU/L)	35.23±2.13	20.60-53.07
Urea	(mg/dl)	30.27±1.84	18.48-45.93
Creatinine	(mg/dl)	1.05±0.11	0.57-2.36

SGOT=Serum glutamic-oxaloacetic transaminse, SGPT=Serum glutamic-pyruvate transaminase, SE=Standard error

### Urine analysis

The urine samples of 10 geriatric dogs were taken for routine pathological examination of urine. Physical examination of urine revealed yellow, amber, red and deep red color, turbidity in 7 cases, little higher specific gravity in 8 cases, Chemical examination revealed positive for protein in 7 cases, glucose in 5 cases, ketone bodies in 3 cases, blood in 3 cases, bilirubin in 4 cases. Microscopic examination revealed no significant finding. The culture and sensitivity test of the same ten urine samples were conducted. Out of them 7 samples showed cultural growth with presence of *Staphylococcus* in 2 cases, *Streptococcus* in 2 cases, and mixed infection with *Streptococcus, Staphylococcus* and *Micrococcus* in 2 cases and *Staphylococcus, Micrococcus* and *Klebsiella* in one case. Antibiotic sensitivity test revealed highest degree of sensitivity to amoxycillin/clavulanate followed by kanamycin, ceftriazone. The result showed the highest degree of resistant to tazobactum and lincomycin.

## Discussion

Ageing represents a complex biological process characterized by a progressive modification of tissues and cells with a gradual loss of adaptive capacity [7]. Diseases associated with advancing age and/or the geriatric life stage include obesity, endocrine dysfunction (such as diabetes and thyroid disease), renal disease, degenerative joint disease, periodontal disease, cardiac disease, behaviour issues and neoplasia [8]. It is a well-established fact that old age or geriatrics is naturally selected and genetically programmed in all species of animals. Old age or geriatrics is a heterogenous process that ultimately leads to the progressive reduction of ability and gradual onset of morbidity. The most common and the first sign of ageing is slowing down. Old age is manifested with change in behavioural pattern to different visible changes.

In the above study, external visible changes revealed graying of hair with a dull appearance of skin and hair coat as finding in old age. In some cases, it was associated with dry skin and graying of hair around eye and muzzle. Old age-related nutritional deficiency might be the cause of this kind of disorder [9]. Dental diseases like dental wear and tear in geriatric dogs were manifested on many occasions. Dental tartars, gingivitis, broken tooth and halitosis were also usual findings in geriatric dogs. This may be attributed to the non-maintenance of proper oral hygiene along with taking of more amount of non-vegetarian diet [10]. Calluses were one of the most common findings in old age mostly in dog’s elbow region. Older dogs are especially prone to calluses on their elbows because of the longer periods they spend lying down. Calluses can also form due to deficiencies in zinc, or as a result of taking calcium supplements, which tends to absorb the mineral zinc. Calluses can also be formed when dogs are overweight or as results of problems in their joints that result in uneven calluses forming on paw pads [9]. Glaucoma was an important finding in older dogs may be arisen due to vitamin deficiency. Due to osteoarthritis many old animals facing difficulty in walking or climbing stair cases. This may be due to articular erosion or change of constituents of the synovial fluid [11] or due to disturbance of calcium and phosphorous metabolism in geriatric dogs [12]. All these visible changes can be considered for correlating with old age.

The hemoglobin, TEC and PCV profile showed a decreasing trend in the geriatric groups. This may be due to decreased bone marrow production, splenomegaly and decreased erythrocyte production. It may also occur due to deficiency of vitamins like copper and zinc in old dogs. Lower value may also be encountered due to chronic kidney disease that results in decreased erythropoietin production and decrease capacity of bone marrow to produce RBC, which ultimately results in lower TEC production. There was lowered hemoglobin, PCV and TEC value in older dogs as per Bhar *et al*. [13]. TLC was increased with higher mean neutrophil count in many cases that might be due to secondary systemic infection.

Biochemical values like total protein, albumin, calcium level showed a decreasing trend while urea level with an increasing trend in geriatric dogs without any much alteration in SGOT, SGPT, cholesterol and craeatinine. Decreased value of albumin concentration with the progressive age are mostly due to liver cirrhosis that alters liver function or malnutrition in geriatric dogs [14]. Decreased total protein value encountered in older dogs might be due to chronic kidney disorder (CKD) and liver disorder in association with malnutrition. There was also marked increase in the urea level in serum in older dogs [14]. The increase in the urea value was perhaps due to progression of kidney disease in old dogs [15]. Routine examination of urine revealed turbidity with high specific gravity in majority of the cases along with proteinuria. Expression of protein in the urine was also typically increased in case of geriatric dogs [16,17]. Kidney dysfunction or nephritis might be the major contributing factor for the proteinuria. The magnitude of proteinuria was 1.5-2 fold of normal or greater which may be due to intake of dietary protein [16]. On microscopic examination, there was the presence of pus cell and epithelial cell in 7 cases and crystals in 4 cases. This indicates kidney dysfunction and urinary infection are common findings in old aged dogs [18,19]. Das [20] studied prevalence of CKD in older dogs with respect to breed and found that CKD was recorded more in large-sized breed (13.4 %) including highest percent of Doberman and lowest in German Spitz. Bacteria like *Streptococcus*, *Staphylococcus* and *Micrococcus*, were the major involved bacteria for the urinary infection in old age in our study. This type of bacteria was also isolated by Kumar *et al*. [21] during old age in dogs. Antibiotic sensitivity test revealed highest degree of sensitivity to amoxycillin/clavulanate followed by kanamycin, ceftriazone.

## Conclusion

External visible changes are still the golden standard of determining the old age in dogs. Hemato-biochemical evaluation can be useful for correlating with the patho-physiological status of the animal. Urine analysis can be employed rightly as kidney dysfunction is a major geriatric problem. Anemia, jaundice, nephritis, hepatitis are the most common gross and microscopic findings observed in geriatric dogs that should be considered during old age. Above all determination of geriatric age and status in dogs through above parameters will help in better clinical management.

## Authors’ Contributions

SP, SKP, APA and SS designed the experiments. SP, MB and SSB carried out the experimental work. SKP, SS, SP and MB were involved in scientific discussion and analysis of the data. SP, MB, SSB, SKP, APA and SS drafted and revised the manuscript. All authors read and approved the final manuscript.
